# How to Access and Use Data From the Wisconsin Longitudinal Study

**DOI:** 10.1093/geroni/igab046.854

**Published:** 2021-12-17

**Authors:** Carol Roan

**Affiliations:** University of Wisconsin - Madison, Madison, Wisconsin, United States

## Abstract

With over 27,000 analysis variables covering more than 60 years of participants’ lives, the WLS data can be overwhelming to new users who are looking for the measures they need to answer their research questions. Core WLS survey data is free and easy to download from our website. As we add new types of measures and new waves of data, we refine our data sharing methods to balance our need to make the data easily available with the need to protect the confidentiality of participants. This presentation will teach users how to access to the data files they need for their research and how to use our online documentation of survey instruments and data files. Symposium attendees will also receive a USB drive with the publicly available data and complete documentation.

